# Disparities in Cancer-Related Avoidable Mortality by the Level of Area Deprivation in South Korea

**DOI:** 10.3390/ijerph18157856

**Published:** 2021-07-25

**Authors:** Woorim Kim, Seongkyeong Jang, Gangeun Lee, Yoon Jung Chang

**Affiliations:** National Cancer Center, National Cancer Control Institute, Division of Cancer Control & Policy, Goyang-si 10408, Korea; wklaura@gmail.com (W.K.); seongkyeong@ncc.re.kr (S.J.); gangeun@ncc.re.kr (G.L.)

**Keywords:** avoidable mortality, area deprivation level, cancer disparity, deprivation, gap

## Abstract

Background: This study investigated trends in cancer-related avoidable (preventable and treatable) mortality and its association with area deprivation in Korea. Methods: Cancer-related avoidable mortality rates per 100,000 population between 2015 and 2019 were measured using the Causes of Death Statistics. Area Deprivation Index (ADI) was measured from the Population and Housing Census and information on other independent variables from the Korea Community Health Survey. The gap in avoidable mortality between the more and less deprived groups was expressed as rate ratios (RR) and absolute differences (ADs) with a 95 percent confidence interval (95% CI). The association between avoidable mortality and ADI was investigated through Poisson regression modelling. Results: The more deprived areas had higher avoidable (RR 1.15, 95% CI 1.13–1.17; AD 6.58, 95% CI 5.59–7.57) and preventable (RR 1.19, 95% CI 1.17–1.21; AD 6.22, 95% CI 5.38–7.06) mortality. The overall cancer-related avoidable mortality decreased but the gap between the more and less deprived groups did not decline significantly during the study period. The association between avoidable and preventable mortality and area deprivation remained significant after adjusting for variables, including area levels of smokers and alcohol drinkers. Conclusions: The gap in avoidable mortality signifies the importance of addressing related disparities in cancer.

## 1. Introduction

Avoidable mortality is defined as deaths that are considered preventable or treatable, based on the availability of current public health and medical interventions [1]. Preventable deaths can be avoided before disease onset through the effective implementation of public health and primary prevention interventions [2]. Treatable mortality refers to deaths that can be prevented or delayed after disease onset through well-timed and effective medical interventions [2]. Deaths that result from preventable causes are associated with reduced incidence and those resulting from treatable causes are associated with decreasing case fatality rates [2]. Therefore, quite predictably, avoidable mortality can be utilized as a public health indicator to assess the performance of a healthcare system or policy, in addition to the identification of areas that require targeted healthcare interventions [3]. In view of the fact that avoidable mortality rates constitute an important component of various health indicators, including life expectancy, the consensus is that effective healthcare systems should focus on minimizing or delaying avoidable mortality [4,5].

Many deaths that result from diseases and injuries, including cancer, are defined as deaths secondary to avoidable causes. Deaths from various types of cancers, including those of the stomach, liver, and lung, which represent some of the most common cancer types in South Korea, are categorized as preventable. Other cancer types, such as colorectal and breast cancer, are classified as treatable. It is important to investigate the factors associated with cancer-related avoidable mortality in view of the significant health and financial burdens imposed by cancer worldwide, accounting for approximately 9.6 million deaths globally and around a quarter of total deaths in Korea, with approximately 36–45% of cancer-induced mortality being classified as avoidable [6,7,8,9]. The economic costs of cancer are significantly high as cancer-related expenditure was approximately USD 125 billion in the United States [10].

The area deprivation level is identified as a contributor to disparities in mortality rates and marginalized populations show a higher risk of adverse health outcomes [11,12]. Similarly, it is known that disadvantaged sections of society endure a disproportionate burden of cancer-related mortality [8]. Although many countries have shown a decrease in the annual number of avoidable deaths, trends in the disparity gap between the high and low socioeconomic groups with regard to cancer survival are unclear [13]. Socioeconomic disparities tend to be particularly greater with regard to preventable mortality, in which a significant number of cancer deaths globally are considered potentially avoidable through the prevention of well-known risk factors, such as smoking, diet, and obesity [14]. However, despite the awareness regarding the importance of reduction in disparities in cancer-related avoidable mortality, few East Asian studies have investigated this subject using large national data. 

In this study, we investigated the overall and annual trends of cancer-related avoidable (preventable and treatable) mortality between 2015 and 2019 in Korea, in addition to the disparity gap between the high and low area deprivation level groups. The association between cancer-related avoidable mortality and the area deprivation index (ADI) was also examined. We hypothesized that cancer-related avoidable mortality will be higher in the more deprived areas.

## 2. Materials and Methods

### 2.1. Data and Study Population

We investigated overall and annual trends of cancer-related avoidable (preventable and treatable) mortality from 2015 to 2019, along with the association between cancer-related avoidable mortality and area deprivation level (ADI) using several data. Specifically, cancer-related avoidable mortality, ADI, and other covariates were measured using data retrieved from several sources for the years 2015 to 2019, which were the most recent five years in which data were available. The unit of analysis was the 253 administrative divisions of Korea. In Korea, municipal level administrative divisions are categorized into city, county, and district level divisions, in which cities exceeding a certain population size are further organized into districts. The average population size for a city was around 328,000, county 54,000, and county 287,000 to 317,000 individuals in 2019 [15].

Different datasets were used to perform the analysis in the following manner: (i) Cancer-related avoidable mortality was calculated using the 2015 to 2019 Causes of Death Statistics. Individual mortality records were extracted to calculate the rates of cancer-related avoidable mortality per 100,000 individuals in the 253 administrative divisions. (ii) The ADI of the 253 administrative divisions was measured using the 2015 Population and Housing Census, which included a representative sample of 2% of the national population. With regard to areas that were further organized into districts, the district-level population was taken as the unit of analysis. Individual or household records of a series of variables were recorded to create and measure the ADI of each division. (iii) Health-related information (smoking, high-risk alcohol consumption, unmet need, and depression) on each of the regional divisions was analyzed based on the 2015 to 2019 Korea Community Health Survey (KCHS). The KCHS was also used to obtain data on the residents’ average length of residence. (iv) Data showing the number of healthcare institutions available in each region were obtained from the Statistics City Yearbook of Korea (SCYK). The Causes of Death Statistics, census data, and SCYK can be used after application and approval by Statistics Korea. Similarly, the KCHS can be downloaded from the website after application and approval. Each dependent or independent variable was analyzed and obtained using the corresponding data detailed above and were combined to form a final dataset organized at the regional division level.

### 2.2. Dependent Variable

The dependent variable was cancer-related avoidable, preventable, and treatable mortality. Cancer-related avoidable mortality is a preventable and treatable cause of mortality. The dependent variable was measured using individual mortality records of those aged between 0 and 74 years, available in the 2015 to 2019 Cause of Death Statistics. The list of causes was based on the preventable and treatable causes of death (2019 version) published by the joint Organisation for Economic Co-operation and Development/European Statistical Office [2]. The list on preventable cancer included lip, oral cavity and pharynx cancer (International Classification of Diseases, 10^th^ Revision (ICD-10) C00-C14), oesophegal cancer (C15), stomach cancer (C16), liver cancer (C22), lung cancer (C33-C34), mesothelioma (C45), skin cancer (C43), bladder cancer (C67), and cervical cancer (C53, 50%) [2]. The list on treatable cancer included colorectal cancer (C18-C21), breast cancer (C50, female), uterus cancer (C54, C55), testicular cancer (C62), thyroid cancer (C73), Hodgkin’s disease (C81), lymphoid leukaemia (C91.0, C91.1), benign neoplasm (D10-D36), and cervical cancer (C53, 50%) [2].

### 2.3. Independent Variable

An interesting variable was the area deprivation level, measured based on the ADI. The ADI was calculated using the Korean version of the ADI developed by Kim (2013), which was used in previous studies [16,17]. The Korean version of the ADI is derived from nine census variables measured in small geographical units, the 253 administrative divisions of Korea. The variables included low socioeconomic status (percentage of workers employed in the agricultural and fishery sectors, self-employed simple laborers, and temporary or day laborers aged between 15 and 64 years) [18], poor living conditions (percentage of households without a modern or individual kitchen, bathroom, bathing facility, water supply, or heating facility), low education level (percentage of individuals aged between 30 and 64 years without a household diploma), no access to a car (percentage of households without car ownership), divorced or widowed individuals (percentage of divorced or widowed individuals aged ≥ 15 years), single-person households (percentage of single-person households), female householders (percentage of households with a female householder), elderly population (percentage of individuals aged ≥ 65 years), and non-apartment residence (percentage of householders not residing in an apartment-type residence). These variables were summed into a composite index through normalization and standardization (z-scores). Based on the calculated ADI, each area was sectored and ranked into quintiles; Q1 refers to the most deprived and Q5, the least deprived area. The Q1 to Q2 groups were categorized as the more deprived area and the Q3 to Q5 groups as the less deprived area. 

### 2.4. Covariates

The following area-level covariates were included in the study: region (metropolitan cities or provincial regions), the residents’ average length of residence (<12 years or ≥12 years) in the regional division, percentage of current smokers (low, low-middle, middle-high, high), percentage of high-risk alcohol consumers (low or high), percentage of the population with an unmet need for healthcare (low or high), percentage of the population with depressive symptoms (low or high), the number of hospitals in the area (below or above average), the number of clinics in the areas (below or above average), and the year (2015 to 2019). The residents’ average length of residence was measured based on the following question: “For how long have you lived in your currently residing administrative division?” The residents’ average length of residence for each regional division was categorized into less than or equal to or above 12 years, the median value of the study sample.

### 2.5. Statistical Analysis

Total and annual cancer-related avoidable, preventable, and treatable mortality per 100,000 population were calculated. Rates were also measured by area deprivation level, categorized into the more (Q1 to Q2) and less (Q3 to Q5) deprived groups. All rates were age-standardized based on the resident registration population of Korea in 2005, which has been applied in previous research [19]. The rate ratio (RR) and absolute difference (AD) with their 95 percent confidence interval (95% CI) between the more and less deprived ADI groups were analyzed to identify the presence of disparity gaps. The *p* for the trend of overall avoidable mortality and the differences between the more and less deprived areas was analyzed. The association between cancer-related avoidable, preventable, and treatable mortality and area deprivation level was determined using Poisson regression modelling. Analysis was conducted with adjustment for all covariates. The SAS software, version 9.4 was used for analysis.

## 3. Results

The total age-standardized annual rates per 100,000 population in years 2015 to 2019 for cancer-related avoidable, preventable, and treatable mortality are presented in Table 1. Cancer-related avoidable mortality was 44.5 (95% CI 44.0–45.0), preventable mortality 33.4 (95% CI 32.9–33.8), and treatable mortality 11.1 (95% CI 10.9–11.3). The more deprived groups had higher avoidable (RR 1.15, 95% CI 1.13–1.17; AD 6.58, 95% CI 5.59–7.57), preventable (RR 1.19, 95% CI 1.17–1.21; AD 6.22, 95% CI 5.38–7.06), and treatable (RR 1.03, 95% CI 1.00–1.07; AD 0.36, 95% CI −0.05–0.77) mortality.

Annual age-standardized annual rates per 100,000 population for cancer-related avoidable, preventable, and treatable mortality are shown in Table 2. Avoidable (*p*-trend 0.0041), preventable (*p*-trend 0.0030), and treatable (*p*-trend 0.0238) mortality revealed a decreasing trend over time. Avoidable and preventable mortality rates were significantly higher in the more than the less deprived group throughout 2015 to 2019 but no statistical significance was found regarding treatable mortality. The difference between the more and less deprived groups did not exhibit a pattern of decrease for cancer-related avoidable (*p*-trend 0.3739), preventable (*p*-trend 0.1387), and treatable (*p*-trend 0.1909) mortality over time.

The results of the analysis on the association between area deprivation level and cancer-related avoidable, preventable, and treatable mortality with adjustment are presented in Table 3. Compared to the less deprived area, the more deprived area had higher avoidable (RR 1.13, 95% CI 1.11–1.15) and preventable (RR 1.16, 95% CI 1.13–1.19) mortality. Treatable (RR 1.03, 95% CI 0.98–1.07) mortality showed a similar tendency but did not have statistical significance.

## 4. Conclusions

This study highlights several important findings. First, overall rates of age-standardized cancer-related avoidable, preventable, and treatable mortality showed a decreasing trend between 2015 and 2019. Second, total and annual rates of age-standardized cancer-related avoidable and preventable mortality differed between the more and less deprived areas. In general, cancer-related avoidable and preventable mortality was significantly higher in the more deprived groups. Third, the disparity gap between the more and less deprived areas in cancer-related avoidable, preventable, and treatable mortality did not exhibit a significantly declining trend during the study period. Fourth, an association was found between cancer-related avoidable and preventable mortality and area deprivation level after adjustment, with more deprived areas having higher avoidable and preventable mortality. Treatable mortality revealed a similar tendency but without statistical significance.

The overall cancer-related avoidable, preventable, and treatable mortality has decreased between 2015 and 2019, which may have partially been impacted by the general reduction in age-standardized cancer mortality in Korea. Specifically, cancer mortality has decreased from around 114 to 73 per 100,000 population between 1999 and 2018 [9]. Yet at the same time, areas with more deprivation showed higher cancer-related avoidable and preventable mortality. A reduction in the disparity gap between the more and less deprived groups during this time period was not found. In relation, previous literature has reported that it is comparatively difficult to achieve a decline in relative mortality because it necessitates interventions that produce a greater effect on the more deprived groups [20]. These findings together suggest improvements in the Korean healthcare system to reduce disparities in cancer-related avoidable mortality.

With regard to the association between cancer-related avoidable mortality and area deprivation level, our findings are in accordance with those of several previous studies that have investigated the effect of socioeconomic inequalities on cancer outcomes. Studies have reported that cancer survival rates are relatively lower in the more deprived areas, even in countries with a comparatively comprehensive universal health insurance system [21]. For example, individuals of low socioeconomic status, who reside in disadvantaged neighborhoods, were shown to have a high risk of cancer mortality in Taiwan [22]. A large study in Germany concluded that compared with other districts, cancer survival was the poorest in the most deprived districts, even after adjustment for cancer stage [23]. Similarly, another study in Germany revealed significantly excess cancer-related avoidable mortality in the more deprived regions [24]. Deprivation was also positively associated with breast cancer survival in England, in which each unit increase in deprivation quintile was associated with an increase in excess mortality [25]. The results of our study make a significant contribution to the existing literature because our data obtained from the general population show that area deprivation levels may also be associated with cancer-related avoidable deaths in Korea. 

One interesting finding of our study is that it reveals that the differences in cancer-related avoidable mortality between the more and less deprived groups are largely related to preventable mortality. Whereas preventable mortality was significantly higher in the more deprived groups, such increases with regard to treatable mortality did not show statistical significance. Disparities in preventable mortality may have been more distinguishable because it includes cancer types that have higher death rates in Korea. For example, lung cancer is known to have the highest and liver cancer the second-highest mortality rate [9]. Stomach cancer was also reported as the second-highest contributor to preventable deaths among Korean women [5]. Such findings on preventable mortality infer the potential importance of health behaviors, including smoking and high-risk alcohol consumption, in addressing disparities between the more and less deprived areas in cancer-related avoidable mortality.

Following are the limitations of this study. First, the study focused on the level of area deprivation, which is an area-based measure of socioeconomic status. Therefore, like all similar measures, the deprivation level applied does not reflect the status of all individuals residing in an area. The fact that the population size of the different regional divisions differs should also be taken into account. Second, the ADI was calculated based on the 2015 census data because these data are released only every five years by Statistics Korea. However, data on mortality and the ot#her covariates were available for all years. Third, several versions are available for the classification of avoidable deaths. This study categorized avoidable mortality based on the preventable and treatable causes of death published by the OECD. Therefore, comparisons with other studies should be performed cautiously. Last, this study was conducted over five years, which is a relatively short time period to assess annual changes in cancer-related avoidable mortality rates. However, despite the limitations stated above, the findings offer important insights as this study is the first to investigate trends in disparities in cancer-related avoidable mortality based on the level of area deprivation in Korea.

In conclusion, a disparity gap was present between the more and less deprived areas in cancer-related avoidable mortality in Korea. Despite overall cancer-related avoidable mortality showing a declining trend between 2015 and 2019, the disparity gap found did not exhibit such tendencies. A significant association was also observed between cancer-related avoidable mortality and area deprivation level, in which avoidable and preventable mortality were higher in the more deprived areas. As health disparity is an important component of public health research and policy investment, further research is warranted to investigate and address disparities related to cancer-related avoidable mortality.

## Figures and Tables

**Table 1:** Age-standardized cancer-related avoidable, preventable, and treatable mortality rates per 100,000 population by area deprivation level.

	Total		Less Deprived		More Deprived		Rate Ratio	95% CI	Absolute Difference	95% CI
	Rate	95% CI	Rate	95% CI	Rate	95% CI				
Avoidable	44.5	(44.0–45.0)	43.4	(42.9–44.0)	50.0	(49.1–51.0)	1.15	(1.13–1.17)	6.58	(5.59–7.57)
Preventable	33.4	(32.9–33.8)	32.3	(31.6–33.1)	38.6	(38.1–39.0)	1.19	(1.17–1.21)	6.22	(5.38–7.06)
Treatable	11.1	(10.9–11.3)	11.1	(10.7–11.5)	11.5	(11.3–11.7)	1.03	(1.00–1.07)	0.36	−(0.05–0.77)

**Table 2:** Annual age-standardized cancer-related avoidable, preventable, and treatable mortality rates per 100,000 population by area deprivation level.

	Total			Less Deprived			More Deprived			Rate Ratio	95% CI		Absolute Difference	95% CI	
	Rate	95% CI		Rate	95% CI		Rate	95% CI							
2015															
Avoidable	49.6	(48.6–50.6)		48.7	(47.6–49.7)		54.8	(53.1–56.5)		1.13	(1.09–1.16)		6.16	(4.29–8.02)	
Preventable	37.8	(36.9–38.8)		36.8	(35.9–37.7)		43.2	(41.6–44.9)		1.18	(1.13–1.22)		6.44	(4.75–8.14)	
Treatable	11.7	(11.3–12.2)		11.9	(11.4–12.3)		11.6	(10.8–12.4)		0.98	(0.91–1.04)		−0.29	−(1.13–0.56)	
2016															
Avoidable	47.1	(45.9–48.3)		45.86	(44.8–47.0)		53.5	(51.2–55.8)		1.17	(1.13–1.21)		7.60	(5.29–9.91)	
Preventable	35.7	(34.7–36.7)		34.5	(33.5–35.4)		41.9	(40.1–43.7)		1.22	(1.17–1.26)		7.43	(5.58–9.29)	
Treatable	11.4	(10.9–11.9)		11.4	(11.0–11.8)		11.5	(10.5–12.6)		1.01	(0.94–1.09)		0.17	−(0.83–1.16)	
2017															
Avoidable	44.2	(43.2–45.3)		43.1	(41.9–44.2)		50.0	(48.1–51.8)		1.16	(1.12–1.20)		6.88	(4.85–8.91)	
Preventable	33.1	(32.2–34.0)		32.1	(31.2–33.0)		38.0	(36.4–39.6)		1.18	(1.13–1.23)		5.89	(4.20–7.57)	
Treatable	11.1	(10.7–11.6)		11.0	(10.5–11.5)		12.0	(11.1–12.9)		1.09	(1.02–1.16)		0.99	(0.07–1.92)	
2018															
Avoidable	41.1	(40.1–42.1)		40.3	(39.2–41.3)		45.9	(44.2–47.7)		1.14	(1.10–1.18)		5.67	(3.76–7.57)	
Preventable	30.5	(29.7–31.3)		29.7	(28.8–30.5)		35.0	(33.6–36.4)		1.18	(1.13–1.23)		5.32	(3.76–6.88)	
Treatable	10.6	(10.1–11.1)		10.6	(10.2–11.0)		10.9	(10.0–11.9)		1.03	(0.96–1.11)		0.34	−(0.59–1.28)	
2019															
Avoidable	40.9	(39.8–41.9)		40.0	(39.0–41.0)		46.0	(44.0–48.0)		1.15	(1.11–1.19)		6.01	(3.99–8.03)	
Preventable	30.1	(29.2–31.0)		29.3	(28.5–30.0)		34.8	(33.1–36.4)		1.19	(1.14–1.24)		5.52	(3.89–7.16)	
Treatable	10.8	(10.3–11.2)		10.8	(10.4–11.1)		11.2	(10.3–12.2)		1.05	(0.97–1.12)		0.49	−(0.39–1.37)	

**Table 3:** The association between cancer-related avoidable, preventable, and treatable mortality and area deprivation level.

	Avoidable Mortality *			Preventable Mortality *			Treatable Mortality *		
	Rate Ratio	95% CI		Rate Ratio	95% CI		Rate Ratio	95% CI	
Area deprivation level									
Less deprived	1.00			1.00			1.00		
More deprived	1.13	1.11	1.15	1.16	1.13	1.19	1.03	0.98	1.07
Region									
Metropolitan cities	1.00			1.00			1.00		
Provincial regions	1.00	0.98	1.03	1.00	0.98	1.02	1.02	0.98	1.06
Residents’ average length of residence									
Less than 12 years	1.00			1.00			1.00		
12 years or above	1.04	1.02	1.06	1.06	1.04	1.09	0.97	0.92	1.01
% of current smokers									
Low	1.00			1.00			1.00		
High	1.03	1.01	1.05	1.04	1.02	1.06	1.00	0.96	1.03
% of high risk alcohol drinkers									
Low	1.00			1.00			1.00		
High	1.05	1.03	1.07	1.04	1.02	1.06	1.09	1.05	1.13
% of unmet need									
Low	1.00			1.00			1.00		
High	0.98	0.96	1.00	0.98	0.96	1.00	0.99	0.95	1.03
% of depressive symptoms									
Low	1.00			1.00			1.00		
High	1.00	0.98	1.02	0.99	0.97	1.01	1.03	0.99	1.06
Number of hospitals									
Equal to or higher than average	1.00			1.00			1.00		
Lower than average	0.98	0.97	1.00	0.98	0.96	1.00	0.99	0.96	1.03
Number of clinics									
Equal to or higher than average	1.00			1.00			1.00		
Lower than average	1.04	1.02	1.06	1.04	1.02	1.07	1.03	0.99	1.07
Year									
2015	1.00			1.00			1.00		
2016	0.97	0.94	0.99	0.96	0.94	0.99	0.98	0.93	1.03
2017	0.89	0.87	0.91	0.87	0.85	0.90	0.95	0.90	1.00
2018	0.82	0.80	0.85	0.80	0.77	0.82	0.91	0.87	0.96
2019	0.83	0.80	0.85	0.80	0.77	0.82	0.93	0.87	0.98

* Results adjusted for all covariates.

## Data Availability

Not applicable.

## References

[1] Zygmunt A., Tanuseputro P., James P., Lima I., Tuna M., Kendall C.E. (2020). Neighbourhood-level marginalization and avoidable mortality in Ontario, Canada: A population-based study. Can. J. Public Health.

[2] Organization for Economic Development and Cooperation (OECD), European Commission (2019). Avoidable Mortality: OECD/Eurostat Lists of Preventable and Treatable Causes of Death (November 2019 Version).

[3] James P.D., Manuel D.G., Mao Y. (2006). Avoidable mortality across Canada from 1975 to 1999. BMC Public Health.

[4] Mays G.P., Mamaril C.B., Timsina L.R. (2016). Preventable Death Rates Fell Where Communities Expanded Population Health Activities Through Multisector Networks. Health Aff..

[5] Bahk J., Jung-Choi K. (2020). The Contribution of Avoidable Mortality to the Life Expectancy Gains in Korea between 1998 and 2017. Int. J. Environ. Res. Public Health.

[6] Jung K.W., Won Y.J., Kong H.J., Lee E.S. (2019). Cancer Statistics in Korea: Incidence, Mortality, Survival, and Prevalence in 2016. Cancer Res. Treat..

[7] Shin H.-Y., Kim J., Lee S., Park M., Park S., Huh S. (2020). Cause-of-death statistics in 2018 in the Republic of Korea. J. Korean Med. Assoc..

[8] Knaul F.M., Arreola-Ornelas H., Rodriguez N.M., Mendez-Carniado O., Kwete X.J., Puentes-Rosas E., Bhadelia A. (2018). Avoidable Mortality: The Core of the Global Cancer Divide. J. Glob. Oncol..

[9] Hong S., Won Y.J., Lee J.J., Jung K.W., Kong H.J., Im J.S., Seo H.G. (2021). Cancer Statistics in Korea: Incidence, Mortality, Survival, and Prevalence in 2018. Cancer Res. Treat..

[10] Mariotto A.B., Yabroff K.R., Shao Y., Feuer E.J., Brown M.L. (2011). Projections of the cost of cancer care in the United States: 2010–2020. J. Natl Cancer Inst..

[11] McCartney G., Popham F., Katikireddi S.V., Walsh D., Schofield L. (2017). How do trends in mortality inequalities by deprivation and education in Scotland and England & Wales compare? A repeat cross-sectional study. BMJ Open.

[12] Commission on Social Determinants of Health (2008). Closing the Gap in a Generation: Health Equity through Action on the Social Determinants of Health.

[13] Ellis L., Coleman M., Rachet B. (2012). How many deaths would be avoidable if socioeconomic inequalities in cancer survival in England were eliminated? A national population-based study, 1996–2006. Eur. J. Cancer.

[14] Brawley O.W. (2011). Avoidable cancer deaths globally. CA Cancer J. Clin..

[15] Ministry of Interior and Safety (2019). Regional Government Administrative Districts and Population Status.

[16] Kim D. (2018). Socioeconomic status, area deprivation and health behavior gaps. Health Welf. Policy Forum.

[17] Kim D., Lee S., Ki M., Kim M., Kim S., Kim Y., Yoon T., Jang S., Jung C., Chae H. (2013). Developing Health Inequalities Indicators and Monitoring the Status of Health Inequalities in Korea.

[18] Yoon T., Moon O., Lee S., Jeong B., Lee S., Kim N., Jhang W. (2003). Differences in Health Behaviors among the Social Strata in Korea. Korean J. Prev. Med..

[19] Choi Y., Kim T., Jang E., Kwon H., Jeon M., Kim W., Shong Y., Kim W. (2014). Standardized Thyroid Cancer Mortality in Korea between 1985 and 2010. Endocrinol. Metab..

[20] Mackenbach J. (2015). Should we aim to reduce relative or absolute inequalities in mortality?. Eur. J. Public Health.

[21] Coleman M.P., Rachet B., Woods L.M., Mitry E., Riga M., Cooper N., Quinn M.J., Brenner H., Esteve J. (2004). Trends and socioeconomic inequalities in cancer survival in England and Wales up to 2001. Br. J. Cancer.

[22] Chang C.M., Su Y.C., Lai N.S., Huang K.Y., Chien S.H., Chang Y.H., Lian W.C., Hsu T.W., Lee C.C. (2012). The combined effect of individual and neighborhood socioeconomic status on cancer survival rates. PLoS ONE.

[23] Jansen L., Eberle A., Emrich K., Gondos A., Holleczek B., Kajuter H., Maier W., Nennecke A., Pritzkuleit R., Brenner H. (2014). Socioeconomic deprivation and cancer survival in Germany: An ecological analysis in 200 districts in Germany. Int. J. Cancer.

[24] Jansen L., Kanbach J., Finke I., Arndt V., Emrich K., Holleczek B., Kajuter H., Kieschke J., Maier W., Pritzkuleit R. (2021). Estimation of the Potentially Avoidable Excess Deaths Associated with Socioeconomic Inequalities in Cancer Survival in Germany. Cancers.

[25] Woods L.M., Rachet B., Morris M., Bhaskaran K., Coleman M.P. (2021). Are socio-economic inequalities in breast cancer survival explained by peri-diagnostic factors?. BMC Cancer.

